# PFAS in Peri-Urban Agricultural Water: Assessing the Hazard Index in an Organic Farming Environment in Maryland, USA

**DOI:** 10.3390/toxics14030245

**Published:** 2026-03-11

**Authors:** Candice M. Duncan, Fatemeh Ghezelsofla, Hlengilizwe Nyoni, Jazmin I. Escobar, Odette Mina

**Affiliations:** 1 Department of Environmental Science and Technology, University of Maryland, College Park, MD 20742, USA; fafagh@umd.edu (F.G.); jesco02@umd.edu (J.I.E.); 2 Institute of Energy and the Environment, Pennsylvania State University, University Park, PA 16802, USA; hvn5148@psu.edu (H.N.); oom5021@psu.edu (O.M.)

**Keywords:** PFAS, hazard index, agricultural environment, farming, stakeholder, health impacts

## Abstract

Global efforts to quantify per- and polyfluoroalkyl substances (PFAS) in irrigation water sources have substantially advanced understanding of their potential impacts on human health. The proposed Hazard Index (HI) tool can be used to assess the health risks of PFAS chemical mixtures. To address potential health impacts, irrigation water samples were collected from two organic farms and analyzed to quantify PFAS under non-ideal agricultural conditions with no known direct PFAS input. Results show perfluorobutane sulfonic acid (PFBS) (37 ng/L), perfluorobutanoic acid (PFBA) (24 ng/L), and perfluorohexanoic acid (PFHxA) (22 ng/L) as the most abundant PFAS compounds at agricultural site 1 (AG1). The HI indicates compliance with perfluorohexane sulfonic acid (PFHxS)_branched (0.39) and non-compliance with PFHxS_linear (1.51) when calculated at AG1. Additional results show the presence of hexafluoropropylene oxide dimer acid (HFPO-DA; GenX; 28 ng/L) at agricultural site 2 (AG2), where no known industrial activity, PFAS-containing compounds (e.g., pesticides) are distributed, or PFAS-related manufacturing facilities exist in the area of influence. The HI indicates non-compliance at AG2 (HI = 2.83) for AG2, with GenX contributing much of the calculated risk. These findings suggest the HI may serve as a useful water health indicator for small sites exhibiting very low PFAS concentrations.

## 1. Introduction

Per- and polyfluoroalkyl substances (PFAS) in water have been described globally over the past few decades [1]. Many studies have described PFAS in groundwater [2,3,4,5], leachate [6,7], rivers [3,4,5,8,9], streams [4,5,8,9], lakes [4,8,9], and wetlands [8,9] under varying environmental conditions. PFAS in agricultural water systems have received an increase in visibility due to the human health implications [9,10,11]. The removal and/or remediation of PFAS in irrigation water remains a challenge as science works to meet increasing demands on natural resources while supporting food production for a growing population. More recently, water used for agricultural irrigation systems has received increased investigation [11,12,13]. Irrigation water for agriculture is a complex system that requires an understanding of the water source (i.e., treated wastewater, rainwater, surface water, groundwater) used in farming. Current models and mechanistic descriptions are used to characterize the transport of PFAS from water to soil and subsequent crops, but many are less predictive in trace-level PFAS-contaminated environments. In an agricultural system designated as organic, the site conditions are significant parameters in understanding how and where PFAS contamination occurs. Quantifying PFAS in agricultural irrigation water is a human and environmental health concern as it provides a pathway to understanding human and biota exposure.

Treated wastewater is one of the most common sources of irrigation water in Maryland [14]. There are more than 70 wastewater treatment plants (WWTP) in Maryland, but current removal technologies are still in development for complete PFAS removal prior to potable use [15,16]. As such, trace concentrations of PFAS may be detected in potable water sources influenced by wastewater treatment plant (WWTP) discharges or located near WWTPs [17]. Few technologies exist to remove PFAS from faucets and showers [18] and large-scale irrigation systems [19]. Two treatment technologies that are effective in removing PFAS for drinking water are granular activated carbon (GAC) filtration and reverse osmosis (RO), which can be applied as part of the filtration step when water is pumped directly from the well [18,20]. These filtration systems often report concentrations at or below the US EPA regulatory values [21] of 4 ppt (parts per trillion) for perfluorooctane sulfonic acid (PFOS) and perfluorooctanoic acid (PFOA) individually, and 10 ppt for perfluorononanoic acid (PFNA), perfluorohexane sulfonic acid (PFHxS), perfluorononanoic acid (PFNA), and hexafluoropropylene oxide dimer acid (HFPO-DA; GenX) individually but not at the desired 0 ppt (parts per trillion; ng/L) MCL (maximum contaminant limit) [22].

PFAS in rainwater have been reported as a potential primary source of contamination [23]. This indicates atmospheric deposition through infiltration as a potentially significant source of PFAS in an agricultural environment. PFAS exposure and contamination via precipitation is a complex process requiring an understanding of PFAS transport in the atmosphere. Infiltration through rain or snow contains varying environmental conditions that would influence the sorption capacity of PFAS binding to soil and plant tissue. The conditions include temperature, wind speed, infiltration speed and quantity, humidity, and presence of particulates in the atmosphere (i.e., PM_2.5_) [24]. Recent studies indicate emissions from aqueous film-forming foam facilities, impurities from manufacturing, and long-range atmospheric transport as primary pathways for atmospheric PFAS contamination [25]. Another study showed how cloud formation and systems can transport PFAS in the atmosphere some distance from the point source [26]. Surface water serves as a local point source of PFAS in agriculture due to proximity to anthropogenic sources. PFAS contamination via surface runoff poses a significant source of PFAS in agricultural settings [27,28,29,30]. In many cases, the PFAS concentrations exceed the US EPA proposed drinking water MCL standards [31]. In an agriculture-dominated watershed, Peter and Lee (2025) reported that the maximum total of PFAS concentration in surface water reached 169.5 ng/L, which was substantially higher than concentrations measured in nearby well water (15.7 ng/L) [29]. More than 90% of groundwater wells in the same watershed exhibited total PFAS concentrations below 5 ng/L [29]. PFAS presence in surface water runoff is also potentially contributed from PFAS-contaminated biosolids used as fertilizers [30,32,33]. Groundwater sources are of particular concern as approximately 23 million households rely on private wells for potable water use. PFAS presence in household products that are disposed of through septic systems is an additional source of PFAS presence in groundwater due to the onsite wastewater treatment system effluent. Recent studies reported that PFAS detection in private wells is a more significant contributor due to the presence of human waste in septic systems than the presence of PFAS in groundwater [34,35].

Current studies demonstrate legacy PFAS (PFOS and PFOA) are reported and frequently detected in many environmental systems and multiple media, including soils, water, and aquatic environments, thus reflecting their historical use and persistence [36,37]. In many cases, PFOS and PFOA direct the chemical analysis results as the most abundant in detection and concentration [38]. In some cases, concentrations are reported to be orders of magnitude higher in soil than in water [39]. As such, baseline concentrations can provide a starting point for understanding the ubiquity of PFAS in organic agricultural settings. The reported concentrations for individual PFAS can also be used to better understand the health implications of exposure through the calculated HI. The U.S. EPA Hazard Index (HI) framework [40] estimates mixture risk by summing compound-specific hazard quotients, where each quotient is calculated as the measured PFAS concentration divided by its corresponding health-based water value/reference concentration for compounds included in the HI framework. The resulting HI is a unitless screening-level metric used to evaluate potential cumulative risk from the included PFAS mixture.

The ubiquity of PFAS in agricultural settings is exacerbated by the knowledge gap of PFAS transport in these complex matrices. Specifically, the identification and quantification of baseline concentrations in an agricultural environment with and without known anthropogenic influences is an impactful objective of the scientific question. Quantifying background concentrations of PFAS in an agricultural setting can lead to decision-making on best practices and procedures for stakeholders as they review the potential impacts of long-term contamination. A site with alternative farming conditions can serve as the environmental conditions to quantify baseline and/or background PFAS concentrations. The organic farming practice is defined herein as an alternative farming method. That is, the site is a non-commercial, organic, open field. The 2022 US Department of Agriculture Census reported 42% of all farms in the US are less than 50 acres (i.e., small) [41]. Conversely, the census reports 42% of all farmlands are more than 5000 acres in size (i.e., large).

In 2024, efforts were made to convene PFAS experts by the National Academies of Sciences, Engineering, and Medicine, tasked with advancing the knowledge on PFAS and creating a list of recommendations designed to aid agricultural stakeholders (i.e., farmers, policymakers, academics, legislators, and community members) in understanding the long-term impacts of PFAS in agriculture [41]. The work presented herein directly addresses the knowledge gap relating to PFAS in irrigation water on agricultural lands. Specifically, the work aims to quantify PFAS in water sources at an organic farm where (1) no known PFAS industry or manufacturing facility is in proximity, (2) no known biosolid applications contribute to PFAS exposure, and (3) no known PFAS-containing pesticides are applied. These factors allow the reported PFAS water concentrations to be classified as background concentrations for select modeling applications [41,42,43,44]. To date, reported PFAS concentrations in water in agricultural lands have largely been impacted by the presence of PFAS in biosolid applications, groundwater (i.e., irrigation water) sources, and potential PFAS-containing pesticide applications [30,32,33]. This work calculates the HI for water collected at 2 organic farms in Maryland to understand the impacts on human health when PFAS concentrations are at or below the regulatory MCL. This work suggests that select short-chain PFAS compounds (e.g., PFBS, PFHxS) can be used to alert stakeholders as to the potential transformation to the more persistent and recalcitrant legacy PFAS compounds (i.e., PFOS, PFOA) in the future of an agricultural system.

## 2. Materials and Methods

### 2.1. Site Description

**Figure 1:**
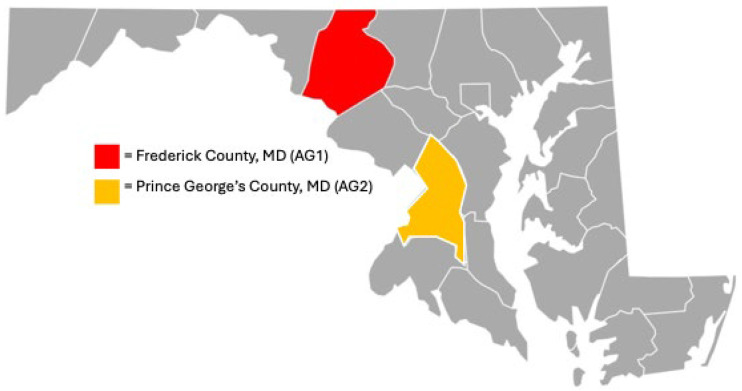
AG1 and AG2 county locations in Maryland, USA.

The primary agricultural site 1 (Figure 1; identified as AG1) is 70 acres with ~30% used for farming in Frederick County, Maryland. The secondary agricultural site 2 (i.e., AG2) is 18 acres with ~55% used for farming in Prince George’s County, Maryland. AG1 is a certified [45] organic practicing farm that maintains its certification annually through the Maryland Department of Agriculture (MDA) and is accredited by the US Department of Agriculture (USDA). AG2 is a “certified naturally grown” farm (since 2013) where weeds and pests are controlled naturally (i.e., flowering farmscapes, cover crops, hand picking). We define non-ideal agricultural conditions as a site with no known biosolids applied and no known PFAS-containing pesticides used. The peri-urban designation is indicative of residential homes bordering both AG1 and AG2 site locations on two or more site perimeters. Each site grows up to 100 different cash crops such as tomatoes, cucumbers, potatoes, and lettuce. More unique items like kohlrabi, Italian sweet peppers, and fennel, along with sought-after herbs and microgreens, are grown.

### 2.2. Water Sampling

**Figure 2:**
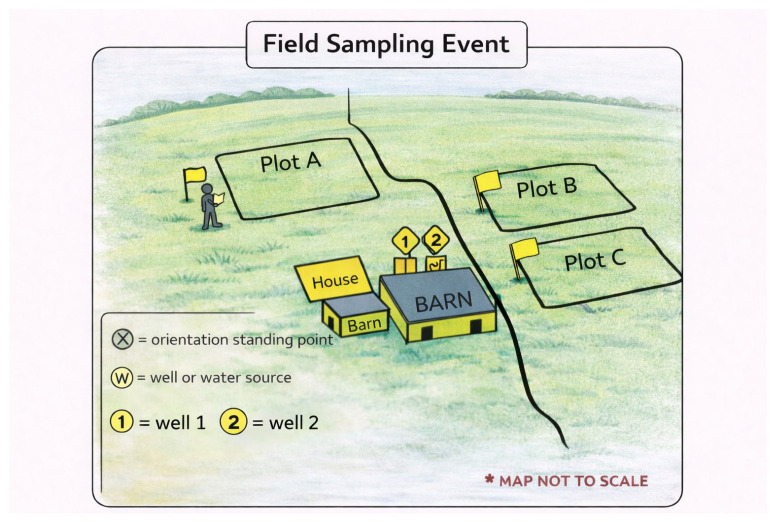
Cartoon of AG1 (created by ChatGPT Version 5.2 from a drawing by Candice M. Duncan).

The AG1 farm uses one well for irrigation plots growing crops. There is one water reservoir (i.e., water holding tank) located onsite directly adjacent to the well. There are 3 hydrants present on the site. Only hydrants 1 and 2 were operable at the time of water sampling in May, June, and August. The well contained a filtration system that permitted filtered water to flow to the hydrants. Filtered and unfiltered well water was collected in June and August of 2025 at AG1. Figure 2 is a cartoon representation of AG1 for illustrative purposes.

The AG2 farm uses one onsite pond for irrigation. As the only irrigation water source, the pond water is filtered using a near-edge pump with a filtration system, and was sampled in August 2025. The exact latitude and longitude (or Google Earth and Maps imagery) of AG1 and AG2 locations are not shared herein due to the potential long-term impacts of the research affecting the livelihood of the farm. Each site provided access to its farm for academic research and informational purposes only. All authors, students, and researchers associated with the project agreed to withhold the site name and location confidentially for these purposes.

### 2.3. Chemicals and Standards

Standards and solvents (i.e., ammonium acetate, acetic acid, methanol, water, acetonitrile, isopropyl alcohol, formic acid) were of high purity and purchased from Fisher Scientific (Pittsburgh, PA, USA). Native PFAS standard (mix) 1633STK (40 analytes); Extraction Internal Standards (24 EIS)—MPFAC-HIF-ES and Non-Extraction Internal Standards (7 NIS)—MPFAC-HIF-IS were purchased from Wellington Laboratories (Guelph, ON, Canada). Polypropylene 0.4 mL autosampler vials with polypropylene caps and pipette tips were from Fisher Scientific (Pittsburgh, PA, USA). Sample bottles and a 10 mL mechanical pipette were supplied by Neta Scientific (Marlton, NJ, USA).

### 2.4. Sample Preparation

#### 2.4.1. Total Suspended Solids (TSS) Pre-Screening

A 100 mL aliquot was collected from the duplicate for TSS analysis. The glass fiber filter was desiccated and weighed, and 30.0 ± 0.02 mL of well-mixed sample was filtered through it. For TSS calculations (mg/L), the filtered volume was converted to liters (0.0300 ± 0.00002 L) to maintain unit consistency. The filter was then dried for a minimum of 12 h at 110 ± 5 °C, cooled in a desiccator, reweighed, and the TSS and percent solids calculated using the Formulas (1) and (2):

TSS (mg/L) = [weight of sample aliquot after drying(mg) − weight of filter (mg)]/0.01 L(1)

% solid = [weight of sample aliquot after drying (g)/weight of sample aliquot before drying (g)] × 100(2)

The measured TSS is used to confirm the suspended solids in an extracted sample with volumes below the reported threshold specified in EPA Method 1633A [46].

#### 2.4.2. Solid Phase Extraction (SPE)

The SPE was performed using Waters Oasis Wax 150 mg Cartridges (WAT-166002493; Milford, MA, USA) equipped with large-volume Sigma-Aldrich SPE reservoirs (25 mL, 54258-U; St. Louis, MO, USA) and placed on a PTFE-free Sigma-Aldrich Visiprep vacuum manifold (57030-U; St. Louis, MO, USA). The samples were homogenized by inverting the sample bottle 3–4 times and allowing it to settle. Each sample bottle was weighed with the lid to 0.1 g, and an aliquot of EIS solution (50 µL) was spiked into the samples. The pH was checked to be 6.5 ± 0.5 and adjusted as necessary with 50% formic acid or ammonium hydroxide (or with 5% formic acid and 3% aqueous ammonium hydroxide). Cartridges were preconditioned by washing with 15 mL of 1% methanolic ammonium hydroxide, followed by 5 mL of 0.3 M formic acid (without vacuum). Each sample was passed through the cartridge at 5 mL/min. The reservoir walls were rinsed with 5 mL of reagent water (twice), followed by 5 mL of 1:1 0.1M formic acid/methanol. The rinsate was drawn through the cartridge under vacuum. The sample bottle was rinsed with 5 mL of 1% methanolic ammonium hydroxide and transferred to the SPE reservoir using a glass pipette. A 25 µL volume of concentrated acetic acid was added to each collection tube and vortexed for 30 s. Approximately 10 mg of activated carbon was added to each sample and intermittently shaken for 2–5 min. The mixture was immediately vortexed (30 s) and centrifuged at 2800 rpm for 10 min. A NIS solution (50 µL) was added to a clean collection tube. A syringe filter (25 mm filter, 0.2 μm nylon membrane) was placed on a 5 mL polypropylene syringe, and the sample supernatant was decanted into the syringe barrel. The plunger was replaced, and the entire extract was filtered into the new collection tube containing the NIS. The mixture was vortexed, and a portion of the extract was transferred to a 1 mL autosampler vial.

### 2.5. Sample Analysis and Instrumentation

#### 2.5.1. Liquid Chromatography

Mobile phase A is 20 mL of PFAS-free Liquid chromatography-mass spectrometry (LC-MS) grade acetonitrile, 20 mL of 100 mM ammonium acetate (prepared by dissolving 770 mg of ammonium acetate in 100 mL of PFAS-free Ultra-High Performance Liquid Chromatography-Tandem Mass Spectrometry (UHPLC-MS) grade water), 1 mL of acetic acid, and 959 mL of PFAS-free LC-MS grade water. Mobile phase B is 20 mL of 100 mM ammonium acetate, 1 mL of acetic acid, and 979 mL of PFAS-free LC-MS grade acetonitrile. The Hypersil GOLD 3.0 × 50 mm, 1.9 um (Thermo Scientific; Pittsburgh, PA, USA) was used as a PFAS delay column, and the Acclaim 120 C18, 2.1 × 100 mm, 2.2 um (Thermo Scientific; Pittsburgh, PA, USA), as an analytical column. The column temperature was maintained at 40 °C. The injected sample volume was set at 5 µL. The Vanquish UHPLC (Thermo Scientific; Pittsburgh, PA, USA) was programmed for chromatographic separation as follows (Table 1):

#### 2.5.2. Mass Spectrometry

Mass spectrometric analysis was performed using a Thermo Scientific TSQ Altis Plus triple quadrupole with a heated electrospray ionization (HESI) source (Thermo Scientific; Pittsburgh, PA, USA) operating in negative-ion mode. Ionization parameters were optimized for stable spray and efficient ion transfer, with a spray voltage of 1500 V, sheath gas pressure at 50 arbitrary units, auxiliary gas pressure at 12 arbitrary units, and sweep gas pressure at 0.5 arbitrary units. The ion transfer tube was maintained at 250 °C, and the vaporizer temperature was set to 225 °C. Data acquisition was performed in timed selected reaction monitoring (SRM) mode, with transitions optimized for each analyte using Thermo’s compound-optimization workflow. The instrument operated with a dwell time based on a 5 s chromatographic peak width, a points-per-peak value of 12.5, a minimum dwell time of 1 millisecond per transition, and a cycle time of 0.4 s. The Argon collision gas was set at 5.5 mTorr, and a resolution of 0.7 FWHM was applied to both Q1 and Q3 quadrupoles to ensure high specificity.

#### 2.5.3. Data Processing and Validation

Data processing followed the methodology described in the documents of McCord et al., 2020 [47]. Initial data (i.e., area counts, retention times, m/z, % area height) were processed in Excel with subsequent statistical analysis performed (e.g., ANOVA, regression statistics). The processing used the guidelines described in Section 11.3 Data Evaluation of the Interstate Technology Regulatory Council [48] and the Data Review and Evaluation.

Guidelines for PFASs Analyzed Using EPA Method 537 [49]. Validation of the analytical method was confirmed by the results reported in the recovery, limit of detection (LOD), limit of quantification (LOQ), method detection limit (MDL), and method quantification limit (Supplementary Materials Table S1). A detailed description of the validation and QA/QC methodology is in the Supplementary Materials Text S1. Data were processed using Thermo Scientific Dionex Chromeleon TM Data System (Version 7.2.10 ES MUg, 27407), and calibration curves were fitted using linear or quadratic regression with 1/x weighting. Section 9 of EPA Method 1633A [46] describes the details of the quality control procedure used in the water analysis. Regression, *p*-value, and subsequent analysis were conducted using RStudio Version 2023.06.1+524 (Copyright (C) 2022 by Posit Software, PBC, Boston, MA, USA).

#### 2.5.4. Data and Statistical Analysis

For AG1, PFAS concentrations were measured at five locations (well non-filtered, well filtered, hydrant 1, reservoir, and hydrant 2). Compounds were grouped into short-chain (i.e., 3 < C < 7) and long-chain (i.e., C > 7), each group analyzed and evaluated separately.

For each compound, concentrations from the five locations were grouped as a single dataset within AG1. Statistics include mean concentration, minimum and maximum concentrations to characterize spatial variability, and cumulative concentration of the five site-specific values. The cumulative concentration was included for descriptive purposes only and was not used in statistical analysis. Compound-specific MDLs were used for reference.

To determine consistent detection above the MDL at AG1, a one-sided, one-sample *t*-test was performed for each PFAS compound. The null hypothesis assumed that the true mean concentration across AG1 equaled the MDL, while the alternative hypothesis assumed the true mean concentration was greater than the MDL.

Multiple compounds were tested within each PFAS group; therefore, *p*-values were adjusted using the Benjamini–Hochberg false discovery rate (FDR) procedure. FDR-adjusted *p*-values less than 0.05 were considered statistically significant. All analyses and figures were generated in R.

#### 2.5.5. Hazard Index Calculation

The Hazard Index is a sum of fractions comparing a PFAS compound measured in the water. The value indicates the highest level acceptable in the absence of health effects. This value can be used to determine compliance at sites where PFAS contamination might pose a significant risk. The Formula (3) below is used to determine the HI [49] at AG1 and AG2:


(3) $$\mathrm{Hazard}\;\mathrm{Index}\;(1\;\mathrm{unitless})\;=\;(\frac{\left[{HFPO-DA}_{ppt}\right]}{\left[10\;ppt\right]})\;+\;(\frac{\left[{PFBS}_{ppt}\right]}{\left[2000\;ppt\right]})\;+\;(\frac{\left[{PFNA}_{ppt}\right]}{\left[10\;ppt\right]})\;+\;(\frac{\left[{PFH\times S}_{ppt}\right]}{\left[10\;ppt\right]})$$

An annual running average value greater than 1.0 is a violation of the proposed HI MCL.

## 3. Results

EPA Method 1633 allows for the quantification of 40 PFAS compounds. This work quantified 14 PFAS compounds across the well (AG1) and pond (AG2) water collected from the agricultural sites in Maryland. The remaining 26 compounds were not detected in the water sampled. The detected compounds were PFBA, PFPeA, PFHxA, PFBS, HFPO-DA (GenX), PFHpA, PFPeS, PFOA_branched, PFOA_linear, PFHxS_branched, PFHxS_linear, PFOS_branched, PFOS_linear, and FOSA_linear (see abbreviations list). Concentrations ranged from 0.5 to 28.3 ng/L (ppt) at AG1 (Table 2) and AG2 (Table 3).

**Figure 3:**
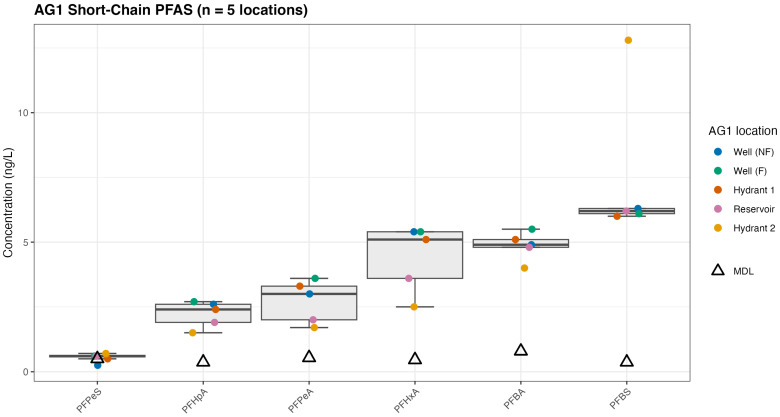
Quantitative short-chain PFAS at AG1: The whisker represents the observed concentration range defined by the minimum and maximum values measured across the five AG1 locations and illustrates spatial variability. It does not represent a confidence interval or statistical uncertainty around the mean.

**Figure 4:**
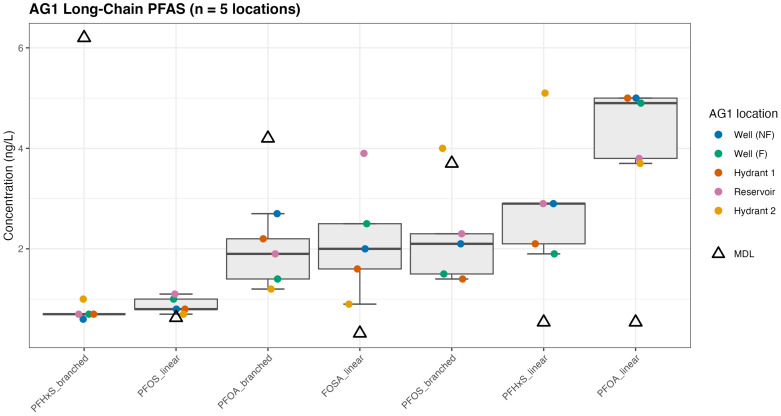
Quantitative long-chain PFAS at AG1: The whisker represents the observed concentration range defined by the minimum and maximum values measured across the five AG1 locations and illustrates spatial variability. It does not represent a confidence interval or statistical uncertainty around the mean.

The range in concentrations of quantifiable short-chain (Figure 3) and long-chain (Figure 4) is shown below for AG1. Each bar represents the range of compound-specific mean values reported at each sampling location for AG1. The triangle represents the MDL, where in some instances, the MDL is higher than the reported concentrations (e.g., PFOA_branched). Concentrations below the MDL, but above the IDL, are both qualitative (detection) and semi-quantitative values (i.e., estimated concentration) reflecting analytical uncertainty for the reported branched compounds.

Short-chain statistical measurements for PFAS at AG1 across five sampling locations (Section 2.5.4) are shown in Figure 3. For the reported short-chain compounds, the mean concentration across AG1 exceeded the compound-specific MDL, except for PFPeS, where most values are at or below the MDL, despite variability among individual locations as indicated by the observed concentration ranges. The short-chain hydrant 2 data represent sampling during the August event only. Hydrant 2 was not operating during the May/June sampling events.

The FDR-adjusted one-sided one-sample *t*-tests comparing the AG1 mean concentration to the MDL indicate PFHpA, PFPeA, PFHxA, PFBA, and PFBS exhibited statistically significant mean concentrations above the MDL (adjusted *p* < 0.05). Results show consistent detection above the MDL when all five AG1 locations are considered together. PFBS indicated the highest mean concentration and the largest cumulative concentration, reflecting both elevated concentrations and spatial variability across AG1. In contrast, PFPeS did not show a statistically significant result (adjusted *p* ≥ 0.05). Although PFPeS was detected at most locations (4/5), its mean concentration across AG1 was negligibly above the MDL.

Long-chain PFAS statistical measurements across the same five sampling locations (Section 2.5.4) at AG1 are shown in Figure 4. Differences were observed between linear and branched isomers with respect to detection above the MDL. The linear isomers PFOS, FOSA, PFHxS, and PFOA exhibited statistically significant concentrations above the MDL (FDR-adjusted *p* < 0.05). PFOA_linear reported the strongest statistical support for detection above the MDL, indicated by the smallest adjusted *p*-value and the highest mean concentration. In contrast, the branched isomers PFHxS, PFOA, and PFOS were not statistically significant (adjusted *p* ≥ 0.05). For these compounds, concentrations were not reliably greater than the MDL. Consequently, the overall AG1 concentrations for branched isomers did not support consistent detection above the MDL. The PFHxS_linear at hydrant 2 and FOSA_linear at the reservoir presented outliers within two of the reported linear isomers.

The AG2 sampling location is a circular pond with one sector serving as an access point to the pump and filtration system. The remaining circumference is another very small, oppositely cleared sector, followed by forested vegetation. PFAS concentrations presented herein are the mean values of one sampling location, as all others are inaccessible.

### 3.1. PFAS Detection

Samples were collected at AG1 in May, June, and August 2025. Samples were collected at AG2 in August 2025 only. Over half of the quantifiable PFAS compounds (57%) are classified as short chain (i.e., 3 < C < 7). The remaining long chain (i.e., C > 7) are primarily PFOS and PFOA, the legacy, recalcitrant, terminal PFAS compounds. Cumulatively (Supplementary Materials Figure S3), PFOS and PFOA are 83% of the long-chain PFAS compounds quantified. The TSS was calculated using Equations (1) and (2). The AG1 TSS values were 0 mg/L for all 5 sampling locations and did not require more than 1 SPE cartridge for sample preparation. The AG2 TSS value is 840 mg/L, thus requiring the use of two or more SPE cartridges for sample preparation prior to analysis.

Current PFAS regulatory concentrations are 4 ng/L for total PFOS and PFOA, respectively. Regulatory concentrations for individual PFNA, PFHxS, and HFPO-DA (GenX) are 10 ng/L. Three sampling locations at AG1 detected PFOA_linear at 5.0, 4.9, 5.0, 3.8, and 3.7 ng/L, all above the MCL of 4 ng/L. The PFOA_branched reported concentrations were below the MDL (4.2 ng/L) at 2.7, 1.4, 2.2, 1.9, and 1.2 ng/L for the non-filtered, filtered, hydrant 1, and reservoir, and hydrant 2 sampling locations, respectively. The PFOS_branched concentrations were also below the MDL (3.7 ng/L) for all sampling locations, excluding hydrant 2 at 4.0 ng/L. PFNA was not detected at AG1 or AG2. The PFHxS_linear concentrations were above the MDL of 0.5 ng/L and below the MCL of 10 ng/L. The PFHxS_branched were significantly lower than the MDL (6.2 ng/L) at 0.6, 0.7 (reported at 3 sampling locations), and 1.0 for the sampling locations at AG1. The PFBS concentrations at AG1 were significantly higher than the MDL of 0.4 ng/L, ranging from 6.0 to 12.8 ng/L. Notably, GenX was not detected at AG1.

As stated in Section 2.2, AG2 has only one onsite pond that serves as the primary irrigation source for the farm. The PFOA_branched was not detected at AG2, and PFOA_linear reported a negligible concentration (1.0 ng/L) slightly above the MDL (0.5 ng/L). Similarly, no detection was reported for PFOS_branched and PFOS_linear, slightly above the MDL (0.6 ng/L) at 0.8 ng/L. PFHxS was not detected at AG2, and PFBS was reported at 1.4 ng/L, 3.5 times above the MDL of 0.4 ng/L. Notably, the GenX concentration at AG2 (i.e., pond) exceeded the MCL at 28.3 ng/L.

Many of the remaining unregulated PFAS (i.e., PFBA, PFPeA, PFHxA, PFHpA, PFPeS, FOSA_linear) were detected at low concentrations with PFPeS negligibly above the reported MDL (Table 2). The cumulative PFOA_linear (23 ng/L) is approximately 2.5 times higher than the PFOA_branched (9 ng/L) concentration. Conversely, the PFOS_linear (4 ng/L) is approximately 2.5 times lower than the PFOS_branched.

### 3.2. PFAS Abundance

A cumulative abundance ranking of PFAS at AG1 shows PFBS (37.3 ng/L) as the most abundant PFAS compound (Supplementary Materials Figure S1), contrary to much literature identifying PFOS and PFOA as the most abundant in concentration and detection at many sites (See Supplementary Materials Figure S1). The decrease in abundance correlates with an increase in chain length as PFBS and PFBA (C = 4), PFHxA and PFHxS_linear (C = 6), and PFOA_linear (C = 8).

A cumulative abundance ranking of PFAS at AG2 shows GenX (28.3 ng/L) as the most abundant PFAS compound (Supplementary Materials Figure S2) present in the pond, with all other reported PFAS concentrations significantly lower (See Supplementary Materials Figure S2). The additional legacy PFAS detected (i.e., PFOS, PFOA) and PFBS are significantly lower than the regulatory values and are 0.8 ng/L, 1.0 ng/L, and 1.4 ng/L, respectively. The GenX concentration (28.3 ng/L) is significantly higher than all reported PFAS concentrations at AG2 and more than double the reported MCL of 10 ng/L.

### 3.3. Hazard Index

**Figure 5:**
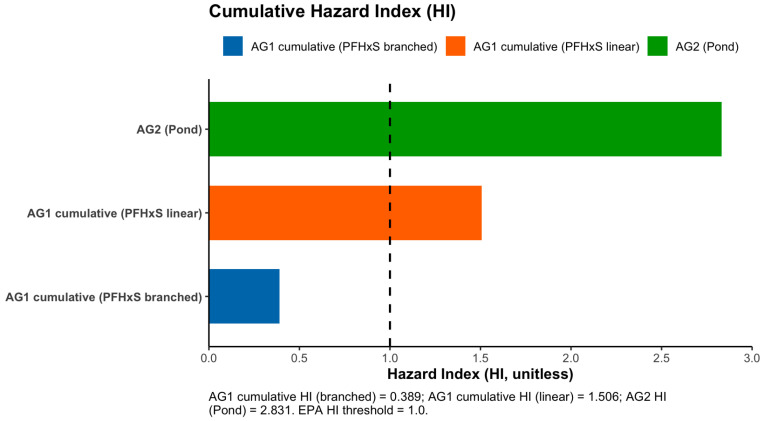
Hazard Index distribution of AG1 cumulative PFHxS_branched, AG1 cumulative PFHxS_linear, and AG2.

The HI (Figure 5) was calculated at five sampling locations at AG1, well filtered, well unfiltered, reservoir, hydrant 1, and hydrant 2. The cumulative HI was calculated using the PFHxS_branched and PFHxS_linear values separately. The HI for AG1 using cumulative GenX, PFBS, PFNA (not detected), and PFHxS_branched is 0.39, indicating compliance with the US EPA value of less than 1.0 (unitless). The HI for AG1 using the cumulative GenX, PFBS, PFNA (not detected), and PFHxS_linear is 1.51, indicating non-compliance with the US EPA value of less than 1.0. The HI for AG2 using the cumulative GenX, PFBS, PFNA (not detected), and PFHxS (i.e., linear or branched; not detected) is 2.83, indicating non-compliance with the US EPA value of less than 1.0.

## 4. Discussion

The results in this work represent two non-ideal agricultural sites. That is, no known biosolids were applied to AG1 or AG2. The farms are certified organic farms reporting no known PFAS-containing pesticides applied and no known industries or municipalities releasing or distributing PFAS-containing water contributing to PFAS in the irrigation systems. As such, the results show PFOS and PFOA, cumulatively, the least abundant PFAS present in both systems (AG1 and AG2). The abundance of PFBS and PFBA in AG1 may indicate the exposure of the precursor 4:2 FtS or 6:2 FtS in the system prior to transformation into PFBA through an aerobic soil microcosm. The grand assumption is that exposure occurred through atmospheric deposition or through subsurface microbial transformation. Additionally, the PFHpA or PFPeA detection can potentially be linked to the transformation from 6:2 FtS based on the proposed transformation pathways in the current literature. The abundance of shorter chain PFAS (i.e., PFBS and PFBA, C = 4; PFHxA, C = 6) can indicate the potential transformation into terminal PFOS or PFOA under these non-ideal environmental conditions. The inverse proportionality of PFOS_branched and linear with the PFOA_branched and linear isomers represents the variability at concentrations close to the MDL and the need to further examine the influence of isomeric structure in irrigation water transport. As research closes the knowledge gap on PFAS in agriculture, stakeholders (i.e., farmers) request an interpretation as to how the concentrations will potentially affect their crops long-term, and less on how PFAS exposure occurred. Identifying and quantifying the PFAS in their irrigation water sources can lead to a better understanding of how to monitor the detected PFAS for the future.

The AG2 sampling location reported low to negligible PFAS concentrations for all reported compounds except for GenX (28.3 ng/L). The results show that GenX present in the pond presents a potential health hazard based on the HI calculation. As a surface water irrigation source, exposure to the surrounding canopy and a visibly present algal layer covering the surface at the time of sampling can potentially explain the transport pathway from the atmosphere to the pond. As a singular sampling event represents a snapshot of PFAS presence in the pond system at AG2, future sampling events will examine and incorporate additional potential factors leading to GenX in the pond. Specifically, longitudinal data of precipitation events, water level fluctuations, and evaporation effects. The reported concentrations represent a starting point for GenX and detectable PFAS monitoring at AG2. It is also noteworthy to identify and attribute groundwater intrusion or additional agricultural inputs as potential PFAS input sources contributing to the high GenX concentration.

In a four-phase study [50] spanning 2020–2022, the Maryland Department of the Environment (MDE; state agency) reported select PFAS concentrations in community water systems (i.e., water treatment plants) and public water systems (i.e., untreated and finished water sources) across the state. In the most proximal wells (*n* = 8) of AG1 (~1.6 km), the total PFOS/PFOA ranged from 1.2 to 8.0 ng/L. The PFBS range was 2.2–4.6 ng/L, and the total PFAS range was 3.5–19.5 ng/L. Values are within an acceptable range at a monitored well in proximity for select compound.

As reported previously, the HI for AG1 used the PFHxS_linear and PFHxS_branched mean values separately. The cumulative HI for PFHxS_linear (1.5 ng/L) at AG1 is approximately four times higher than the cumulative HI value for PFHxS_branched (0.39 ng/L). This indicates that the physicochemical characteristics of each compound can potentially determine the long-term hazard to human health. Specifically, the branched structure has greater mobility in water with limitations in soil sorption due to the exposure pathway (i.e., through a manufacturing impurity or as a designated PFOS replacement). The resultant mobility of branched PFHxS can influence bioaccumulation in human systems. Conversely, the linear PFHxS has a greater affinity for the aqueous solution, potentially affecting the aquatic ecosystem. Additionally, the cumulative concentration of PFHxS_linear for AG1 is 14.9 ng/L and exceeds the MCL of 10 ng/L individually. The equivalent chain length (C = 6) for the branched and linear PFHxS isomers is excluded as the driving factor for abundance, as both contain equivalent molecular formulas. Therefore, environmental conditions and physicochemical properties determine recalcitrance in this system.

The groundwater source of AG1 and the surface water source of AG2 present 2 PFAS exposure pathways, leading to conclusions relating to the monitoring of PFAS at these respective sites. The HI is a valuable screening level tool describing how select PFAS in an agricultural system can potentially impact human health effects long-term. Additional laboratory studies monitoring branched versus linear transport in PFHxS via column experiments in an environmental water system can aid in characterizing the transport of these isomers. Further examination of branched and linear PFAS isomers in irrigation water may improve understanding of isomer-specific transport behavior in these complex systems.

## 5. Conclusions

This study quantified PFAS occurrence and associated health risks in irrigation water from two certified organic agricultural sites operating under non-ideal conditions, where no known biosolids application, PFAS-containing pesticides, industrial activity, or municipal PFAS inputs were reported. Multiple PFAS compounds were detected at both sites, indicating that agricultural irrigation systems can be affected by diffuse PFAS inputs. Across both sites, PFOS and PFOA were among the least abundant compounds, while shorter-chain PFAS were more prevalent, particularly at AG1, suggesting varying transport mechanisms influenced PFAS composition in irrigation water.

Hazard Index (HI) calculations revealed site and isomer-specific differences in potential health risk. At AG1, the HI indicated screening-level HI values above 1 for linear PFHxS but remained compliant for branched PFHxS, indicating that physicochemical differences between isomers influence mobility, persistence, and risk characterization. The elevated concentration of PFHxS_linear relative to regulatory thresholds further highlights the importance of isomer-resolved analysis in PFAS risk assessments. At AG2, GenX (HPFO-DA) was the primary contributor to HI non-compliance, suggesting that surface water irrigation sources may be particularly susceptible to PFAS inputs via atmospheric transport and surface water processes. The reported data highlights a seasonal compliance or non-compliance, indicating additional data is needed for the annual compliance calculation.

Comparison with regional PFAS monitoring data from nearby community and public water systems indicates that concentrations measured in this study are consistent with values reported elsewhere in Maryland, despite differences in water source type and site characteristics. This alignment suggests potential relevance beyond the study sites, while requiring confirmation in other agricultural settings, and indicates low-level PFAS contamination in irrigation water may be more widespread than previously recognized.

Overall, this work indicates that the Hazard Index is a useful screening tool for assessing PFAS mixture risks in agricultural irrigation waters with low-level contamination. The results emphasize the need for routine monitoring of irrigation water sources and for greater attention to PFAS isomer composition in exposure and risk assessments. As this study is based on two sites and a limited sampling framework, the findings should be interpreted as site-specific and exploratory. Continued investigation into PFAS transport, transformation, and long-term behavior in agricultural environments is essential to improve risk management strategies and to inform stakeholders about potential implications for crop production and human health.

## Figures and Tables

**Table 1:** Vanquish UHPLC gradient program for water analysis.

Time (min)	Mobile Phase A (%)	Mobile Phase B (%)	Flow Rate (mL min^−1^)
0.0	90	10	0.4
1.0	70	30	0.4
5.0	54	46	0.4
10.0	24	76	0.4
10.5	14	86	0.4
12.5	14	86	0.4
12.9	90	10	0.4
15.0	90	10	0.4

**Table 2:** Mean range of detected PFAS compounds at AG1.

Compounds	Chain Length	MDL	Mean Range (ng/L)
PFBA	C = 4	0.79	4–5.5
PFPeA	C = 5	0.54	1.7–3.6
PFHxA	C = 6	0.46	2.5–5.4
PFBS	C = 4	0.37	6.0–12.8
HFPO-DA (GenX)	C = 6	0.51	ND
PFHpA	C = 7	0.37	1.5–2.7
PFPeS	C = 5	0.50	ND–0.7
PFOA_branched	C = 8	4.20	1.2–2.7
PFOA_linear	C = 8	0.54	3.7–5.0
PFHxS_branched	C = 6	6.20	0.6–1.0
PFHxS_linear	C = 6	0.54	1.9–5.1
PFOS_branched	C = 8	3.70	1.4–4
PFOS_linear	C = 8	0.63	0.7–1.1
FOSA_linear	C = 8	0.32	0.9–3.9

**Table 3:** Detected PFAS compounds at AG2.

Compounds	Chain Length	MDL	Mean Concentration (ng/L)
PFBA	C = 4	0.79	2.1
PFPeA	C = 5	0.54	1.9
PFHxA	C = 6	0.46	1.1
PFBS	C = 4	0.37	1.4
HFPO-DA (GenX)	C = 6	0.51	28.3
PFHpA	C = 7	0.37	0.8
PFPeS	C = 5	0.50	ND
PFOA_branched	C = 8	4.20	ND
PFOA_linear	C = 8	0.54	1.0
PFHxS_branched	C = 6	6.20	ND
PFHxS_linear	C = 6	0.54	ND
PFOS_branched	C = 8	3.70	ND
PFOS_linear	C = 8	0.63	0.8
FOSA_linear	C = 8	0.32	0.9

## Data Availability

The original contributions presented in this study are included in the article and Supplementary Materials. Further inquiries can be directed to the corresponding author.

## Supplementary Materials

The following supporting information can be downloaded at: https://www.mdpi.com/article/10.3390/toxics14030245/s1, Text S1: Data validation, QA/QC, and LOQ; Table S1: Acceptable limits for the target analytes and pooled ongoing precision recovery for water samples; Figure S1: Abundance ranking of PFAS compounds at AG1; Figure S2: Abundance ranking of PFAS compounds at AG2; Figure S3: Cumulative PFAS concentrations at AG1; Table S2: Cumulative concentrations for hazard index (HI) calculation at AG1.

## Abbreviations

The following abbreviations are used in this manuscript:

PFAS	Per- and polyfluoroalkyl substances
AG1	Agricultural site 1
AG2	Agricultural site 2
PFBS	Perfluorobutane sulfonic acid
PFHxS	Perfluorohexane sulfonic acid
PFNA	Perfluorononanoic acid
PFOA	Perfluorooctanoic acid
PFOS	Perfluorooctane sulfonic acid
GenX	Hexafluoropropylene oxide dimer acid
GAC	Granular activated carbon
RO	Reverse osmosis
WWTP	Wastewater treatment plant
MCL	Maximum contaminant level
HI	Hazard index
MDA	Maryland Department of Agriculture
USDA	U.S. Department of Agriculture
TSS	Total suspended solids
SPE	Solid-phase extraction
NIS	Non-extraction internal standards
LC–MS	Liquid chromatography–mass spectrometry
UHPLC–MS/MS	Ultra-high-performance liquid chromatography
HESI	Heated electrospray ionization
SRM	Selected reaction monitoring
LOD	Limit of detection
LOQ	Limit of quantification
MDL	Method detection limit
MQL	Method quantification limit
