# Supplementary material for: PFAS in Peri-Urban Agricultural Water: Assessing the Hazard Index in an Organic Farming Environment in Maryland, USA
Source: Toxics. 2026 Mar 11;14(3):245. doi: 10.3390/toxics14030245 (PMC13030358; doi:10.3390/toxics14030245)
Supplement: Supplementary file 1 [file toxics-14-00245-s001.zip › toxics-4156581-supplementary.pdf]

Supplementary Materials for Toxics Journal

Article

PFAS in Peri-Urban Agricultural Water: Assessing the Hazard Index in an Organic Farming Environment in Maryland, USA

Candice M. Duncan [1,*], Fatemeh Ghezelsofla [1], Hlengilizwe Nyoni [2], Jazmin I. Escobar [1] and Odette Mina [2]

[1] Department of Environmental Science and Technology, University of Maryland, College Park, MD 20742, USA; fafagh@umd.edu (F.G.); jesco02@umd.edu (J.I.E.)
[2] Institute of Energy and the Environment, Pennsylvania State University, University Park, PA 16802, USA; hvn5148@psu.edu (H.N.); oom5021@psu.edu (O.M.)
* Correspondence: cduncan1@umd.edu; Tel.: +1-301-405-1351

This SI includes one text, two tables, and three figures as supplementary materials, data, and discussions.

Supplementary Materials Text S1: Data Validation, QA/QC, and LOQ

Data validation and processing require the use of quality assurance and quality control methods (QA/QC). This section of the supplemental data is described herein.

The MDL calculation gives the concentration of the analyte that yields a response equal to twice the average baseline noise. The MDL for any instrument is determined after any major changes to the hardware of the instrument, major changes to the method, a new column, or annually. The resultant MDL must be examined for validity. The standard must produce a standard deviation high enough that the resulting MDL is >10% of the average result for the standard. If the MDL is not within expected values, new standards should be made and reanalyzed. This process should be repeated until the MDL results are within the accepted values.

The limit of quantitation (LOQ) reporting limit should be less than the lowest standard. The LOQ is determined experimentally by analyzing dilutions of low-level standards in quadruplicate and establishing the lowest concentration that can be quantified with accuracy and reproducibility.

Quantification requires the creation of a calibration curve. The calibration curve defines the concentration range within which sample concentrations should fall for reliable quantification. The stock solution can be made by calculating ($C1 \times V1 = C2 \times V2$) the volume needed for a six-point calibration curve given a commercially supplied standard solution. These standards are used during all sample analysis, and after every seven samples, the midpoint standard should be run for instrument performance checks. Each calibration standard is analyzed three times, and the area response is tabulated against the mass concentration injected. The results are used to prepare a calibration curve. The slope of the calibration curve gives the response factor, RF. The linear response is indicated where a correlation coefficient of ~0.999 for a linear least-squares fit of the data (mass concentration versus area response) is obtained. The daily response for the analytes of interest should be within 10% of the calibration value. If greater variability is observed, prepare a fresh calibration check standard. If the variability using a freshly prepared calibration check standard is greater than 15%, a new calibration curve must be developed from fresh standards. A plot of the daily values on a Quality Control Chart covering total analysis time (days versus concentrations) is helpful to check for long-term drift of the standard concentration value. Ongoing precision recovery (OPR) summary statistics in Supplementary Materials Table 1 are reported as pooled analyte-specific means and standard deviations across project OPR events (n = 19). Acceptance limits (minimum and maximum OPR %) were based on EPA Method 1633A / laboratory QA/QC criteria. No outliers were excluded unless supported by documented analytical/instrumental error. Elevated standard deviations for some analytes may reflect analyte-specific recovery variability across OPR events.

Supplementary Materials Table S1: Acceptable limits for the target analytes and pooled ongoing precision recovery for water samples.

	Acceptance Limits OPR %		Project % Recoveries (n =19)	
**Compounds**	**Min**	**Max**	**Mean**	**Std Dev**
11Cl-PF2OUdS (F-53B Minor)	55	160	78	33
3:3 FTCA or FPrPA	65	130	97	27
4:2 FTS	70	145	105	10
5:3 FTCA or FPePA	70	135	109	10
6:2 FTS	65	155	106	8
7:3 FTCA or FHpPA	50	145	114	29
8:2 FTS	60	150	108	33
9Cl-PF3ONS (F-53B Major)	70	155	87	26
ADONA	65	145	97	13
FOSA_branched	70	145	103	27
FOSA_linear	70	145	104	7
HFPO-DA (GenX)	70	140	103	7
NEtFOSA_branched	65	145	109	22
NEtFOSA_linear	65	145	101	8
N-EtFOSAA_branched	70	145	169	147
N-EtFOSAA_linear	70	145	107	8
NEtFOSE_branched	70	135	125	45
NEtFOSE_linear	70	135	102	28
NFDHA or 3,6-OPFHpA	50	150	104	13
NMeFOSA_branched	50	150	105	32
NMeFOSA_linear	50	150	105	18
N-MeFOSAA_branched	50	140	91	47
N-MeFOSAA_linear	50	140	103	9
N-MeFOSE_branched	70	145	122	43
N-MeFOSE_linear	70	145	101	8
PFBA	70	140	104	8
PFBS	60	145	85	18
PFDA	70	140	102	7
PFDoA	70	140	103	9
PFDoS	50	145	71	39
PFDS	60	145	86	28
PFEESA	70	140	101	14
PFHpA	70	150	104	7
PFHpS	70	150	115	33
PFHxA	70	145	105	8
PFHxS_branched	65	145	106	13
PFHxS_linear	65	145	105	9
PFMBA or PF5OHxA	60	150	108	9
PFMPA or PF4OPeA	55	140	94	19

PFNA_branched	70	150	107	13
PFNA_linear	70	150	104	7
PFNS	65	145	94	18
PFOA_branched	70	150	112	29
PFOA_linear	70	150	103	8
PFOS_branched	55	150	113	22
PFOS_linear	55	150	102	11
PFPeA	65	135	91	19
PFPeS	65	140	93	22
PFTeDA	60	140	103	11
PFTrDA	65	140	150	60
PFUdA	70	145	103	8

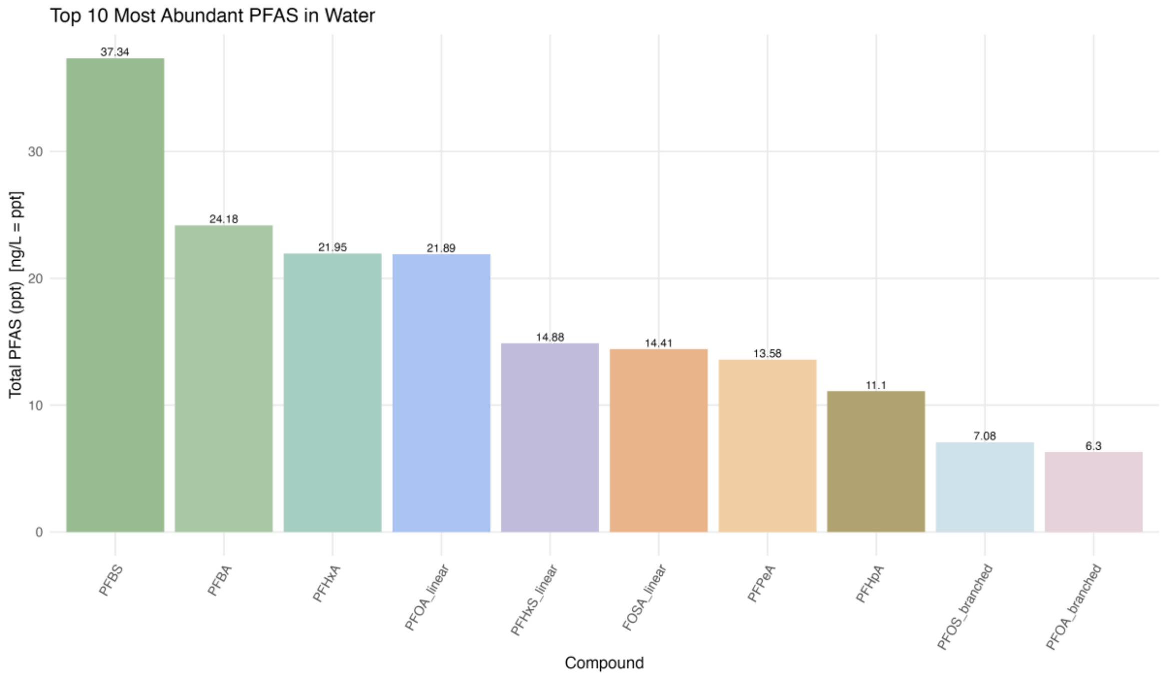

Supplementary Materials Figure S1: Abundance ranking of PFAS compounds at AG1.

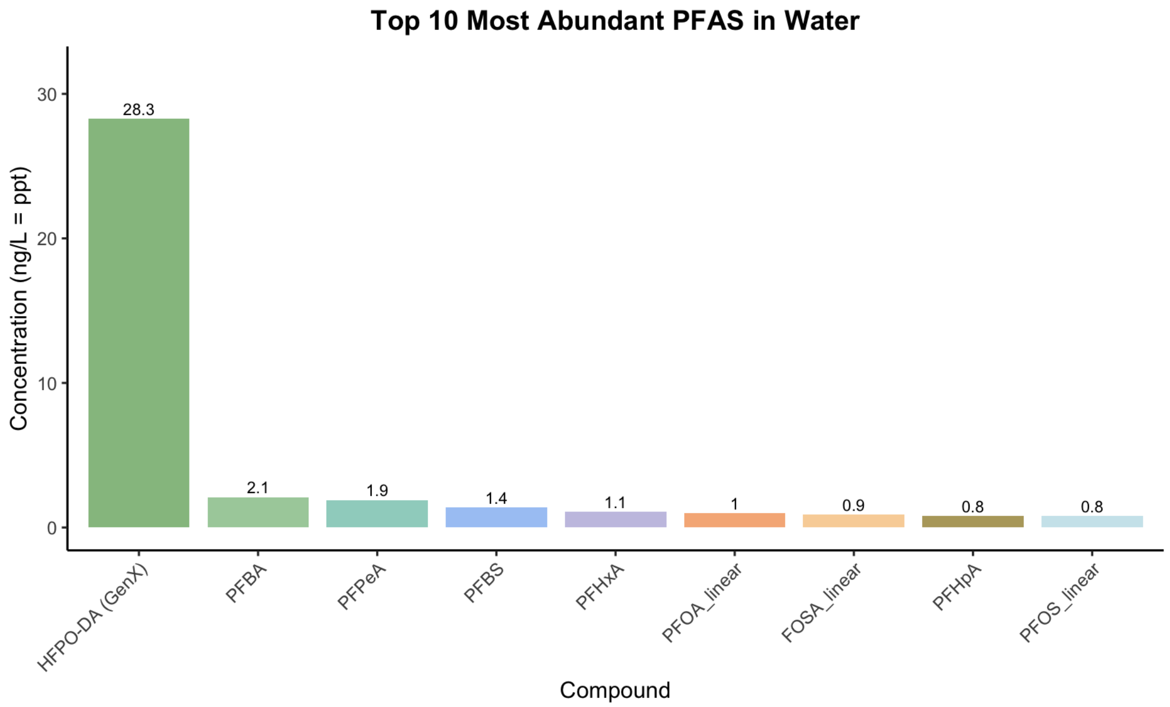

Supplementary Materials Figure S2: Abundance ranking of PFAS compounds at AG2.

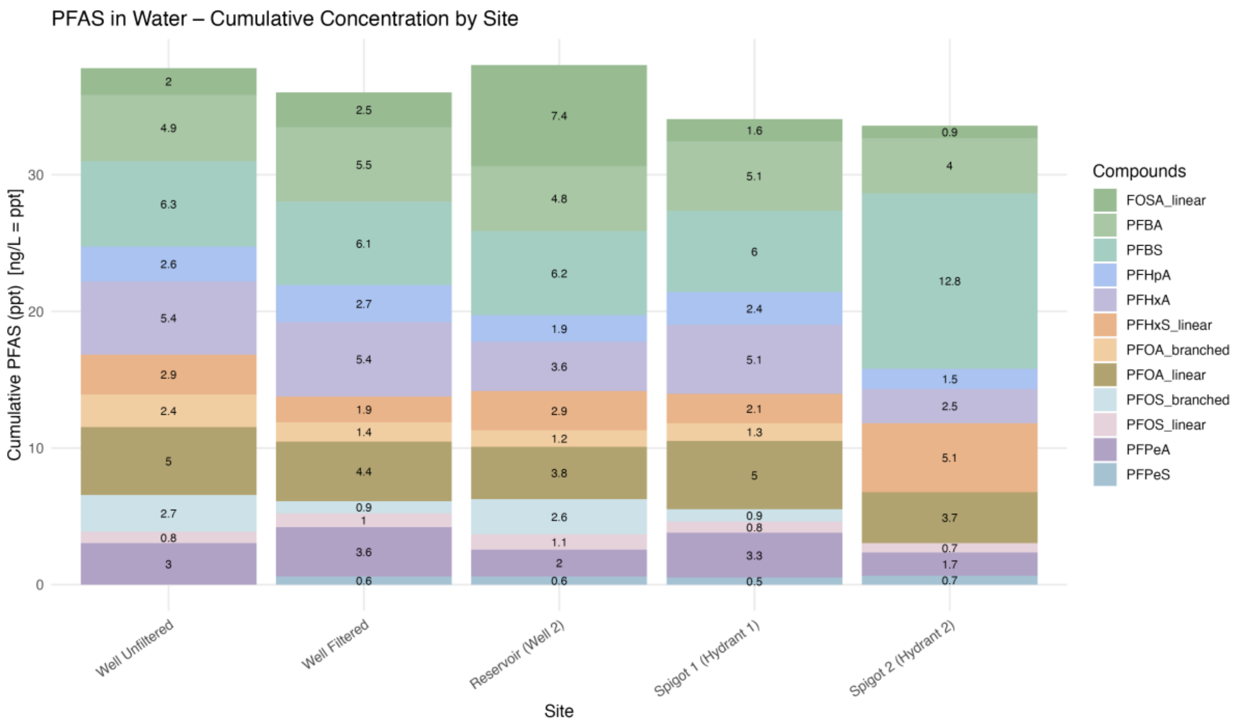

Supplementary Materials Figure S3: Cumulative PFAS concentrations at AG1.

Supplementary Materials Table S2: Cumulative concentrations for hazard index (HI) calculation at AG1. All ND were replaced with zero for the HI calculation.

AG1 (Five locations: Well NF, Well F, Hydrant 1, Reservoir, Hydrant 2)

Compound (ng/L)	Well NF	Well F	Hydrant 1	Reservoir	Hydrant 2	Cumulative concentration
GenX	0.0	0.0	0.0	0.0	0.0	0.0
PFBS	6.3	6.1	6.0	6.2	12.8	37.4
PFHxS (linear)	2.9	1.9	2.1	2.9	5.1	14.9
PFHxS (branched)	0.6	0.7	0.7	0.7	1.0	3.7
PFNA	0.0	0.0	0.0	0.0	0.0	0.0
